# Host-adapted probiotic potential of *Ligilactobacillus agilis* 2-2 revealed by comparative genomic and phenotypic analyses

**DOI:** 10.1016/j.psj.2025.106274

**Published:** 2025-12-12

**Authors:** Zhen Zhang, Yang Lv, Zisheng Guo, Lei Liu, Xiaohui Chen, Wenjing Han, Jinshuo Wei, Songtao Guo, Yanmei Sun, Shiwei Wang

**Affiliations:** a Key Laboratory of Resource Biology and Biotechnology in Western China, Ministry of Education, Provincial Key Laboratory of Biotechnology, College of Life Sciences, Northwest University, 229 Taibai North Road, Xi’an, Shaanxi 710069, China; b Institute of Biomedical Research, Henan Academy of Sciences, Zhengzhou 450002, China; c Shaanxi Key Laboratory for Animal Conservation, College of Life Sciences, Northwest University, Xi’an, Shaanxi 710069, China; d Jian Yang City Product Quality Supervision & Testing Institute, Jianyang, China

**Keywords:** *Ligilactobacillus agilis*, Pan-genome analysis, Host-specific adaptation, Probiotic potential, Carbohydrate metabolism

## Abstract

Host-specific adaptation shapes the evolution of safe and effective probiotics. In this study, we performed the first pan-genome analysis of *Ligilactobacillus agilis* using 40 genomes from poultry and mammalian sources. The species exhibits an open, highly plastic genome with host-driven divergence in carbohydrate metabolism. Glycosyltransferase GT2, *bg*lF_2 and *tcy*B/C were enriched in mammalian strains, whereas capsule biosynthesis gene epsH and *asp*2 were predominant in poultry strains. The poultry-derived strain L. *agilis* 2-2 harbors gene clusters associated with acid and bile tolerance (*atp*A–*atp*H), adhesion (*map*A), short-chain fatty acid biosynthesis (*ldh*A, *ack*A–*pta*), and antioxidant defense (*trx*A/B, *msr*A/B), collectively supporting its intestinal adaptation and probiotic fitness. Phenotypically, L. *agilis* 2-2 exhibited strong acid (86.9 %) and bile (84.1 %) tolerance, high aggregation (75 %) and hydrophobicity (55.3 %), and potent antimicrobial activity, facilitating gut colonization. Its cell-free supernatant displayed strong antioxidant capacity, scavenging DPPH (79.5 %), hydroxyl (66.2 %), and ABTS⁺ (83.2 %) radicals, and produced abundant lactic acid (7.7 mg/mL), butyrate (1767.2 μg/mL), and propionate (1097.0 μg/mL). Collectively, these findings establish L. agilis 2-2 as a metabolically versatile, host-adapted, and genomically safe probiotic, highlighting its potential for targeted poultry applications and providing mechanistic insights into host-specific adaptation in *Ligilactobacillus*.

## Introduction

The gut microbiota plays a pivotal role in host health, contributing to nutrient metabolism, immune regulation, epithelial barrier integrity, and pathogen exclusion (Neurath et al., 2025; Carolak et al., 2025). In poultry, a balanced gut microbiome is strongly associated with enhanced growth performance (Zhang et al., 2022), improved feed efficiency (Wen et al., 2021), and resistance to enteric diseases such as necrotic enteritis and coccidiosis (Broadwater et al., 2025). However, intensive farming practices, characterized by high stocking densities and increased pathogen exposure, frequently disrupt microbial homeostasis and thereby impair gut function and productivity (Li et al., 2023). Although antibiotic growth promoters (AGPs) were historically used to sustain gut health and performance, their role in driving antimicrobial resistance has led to regulatory restrictions and prompted the search for safe and sustainable alternatives (Chatterjee et al., 2019).

Probiotics are increasingly regarded as effective alternatives to AGPs because of their capacity to modulate gut microbiota, enhance mucosal immunity, and inhibit colonization by enteric pathogens (Ohimain et al., 2012). Nevertheless, probiotic efficacy varies substantially among strains and is strongly influenced by their genetic background and compatibility with the host environment. Host-adapted strains, isolated from the same or closely related species, generally exhibit superior colonization efficiency and functional integration within the gastrointestinal tract. Therefore, the targeted isolation and systematic evaluation of candidate probiotics from the intended host species are critical for ensuring ecological adaptation and maximizing functional performance, particularly in livestock systems characterized by species-specific diets, gut physiology, and microbial communities.

*Ligilactobacillus agilis* is a facultatively heterofermentative lactic acid bacterium that was reclassified from the genus *Lactobacillus* following recent taxonomic revisions supported by whole-genome phylogenetic analysis (Zheng et al., 2020). This species has been isolated from diverse ecological niches, including the gastrointestinal tracts of pigs, rats, and poultry, demonstrating its adaptability to different host environments. Preliminary studies suggest that *L. agilis* exhibits probiotic properties, such as antimicrobial activity and moderate tolerance to gastrointestinal stress (Yin et al., 2024; Vezina et al., 2021). However, the functional features of *L. agilis*, especially at the genomic level, remain largely unexplored. Poultry-derived strains have received little attention, even as demand grows for host-adapted probiotics to enhance avian gut health and productivity. Given the host- and strain-specific nature of probiotic efficacy, a systematic evaluation of poultry-derived *L. agilis* integrating genomic, ecological, and functional analyses is essential to assess its potential as a next-generation probiotic for poultry.

To address this gap, integrated whole-genome and comparative genomic analyses provide an effective strategy for identifying safe and functional probiotic strains for both human and animal applications. Effective probiotics must endure gut transit, colonize the host, inhibit pathogens, and remain free of resistance or virulence genes (Yang et al., 2023). Advances in genome sequencing now enable simultaneous assessment of taxonomic identity, metabolic potential, and biosafety risks, including antibiotic resistance genes (ARGs) and virulence determinants relevant to food and health safety (Kingkaew et al., 2024). Collectively, genome-based comparative approaches advance the evaluation of probiotic functionality and safety, supporting the rational design of next-generation probiotics (Çetin and Aktaş, 2024).

In this study, we isolated a novel *L. agilis* 2-2 from a healthy chicken and performed whole-genome sequencing with pan-genome analysis to investigate its genomic and functional traits. The analysis revealed clear host-specific genomic adaptations, with marked differences between poultry- and mammal-derived strains. We further characterized strain 2-2 through *in vitro* assays assessing acid and bile tolerance, antimicrobial activity, and short-chain fatty acid production, all key features of probiotic efficacy in poultry. To our knowledge, this is the first comprehensive genomic and phenotypic characterization of *L. agilis,* providing insight into host adaptation and supporting the development of targeted probiotics to improve poultry gut health and disease resistance.

## Materials and methods

### Isolation, cultivation, and genome-based identification

Strain 2-2 was isolated from the intestinal contents of a healthy chicken by serial streaking on MRS agar at 37°C and stored in MRS broth with 15 % glycerol at −80°C. Genomic DNA was extracted from MRS cultures using a commercial kit (Tiangen, China). The nearly full-length 16S rRNA gene was amplified with primers 27F/1492R, sequenced, and analyzed via EZBioCloud for phylogenetic placement (Yoon et al., 2017). Closely related sequences were aligned with Clustal Omega (Sievers et al., 2011), and neighbor-joining (Saitou and Nei, 1987) and maximum-likelihood (Felsenstein, 1981) trees were constructed in MEGA X (Kumar et al., 2018) with 1,000 bootstrap replicates (Felsenstein, 1985).

Genome-level identification employed ANI, AAI, and dDDH analyses using FastANI (Jain et al., 2018), the AAI calculator (http://enve-omics.ce.gatech.edu/aai/), and GGDC 3.0 (http://ggdc.dsmz.de/distcalc2.php). Orthologous gene clusters were identified using OrthoFinder v2.5.2 (Emms and Kelly, 2019), aligned with Clustal Omega, and concatenated. Poorly aligned regions were trimmed with trimAl (Capella-Gutiérrez et al., 2009), and a maximum-likelihood phylogenomic tree was inferred using IQ-TREE (Nguyen et al., 2015) with 1,000 ultrafast bootstraps. ModelFinder (Kalyaanamoorthy et al., 2017) selected the best-fit substitution model.

### Genome and pan-genome analysis of strain 2-2

Genomic sequencing of strain 2-2 was performed on the Illumina HiSeq X Ten platform (Majorbio BioTech Co., Ltd., Shanghai, China) using 2 × 150 bp paired-end libraries with an average insert size of approximately 400 bp. *De novo* assembly of the genome was performed using SOAPdenovo v2.04 (https://omictools.com/soapdenovo-tool), and remaining assembly gaps were closed with GapCloser (https://sourceforge.net/projects/soap-denovo2/files/GapCloser/). Strain 2-2 was sequenced at an average depth of 683 ×, ensuring high-quality assembly. The genome has been deposited in GenBank (JBSRNI000000000). For comparative analysis, 47 closely related genomes were retrieved from the NCBI database and quality-filtered using CheckM v1.1.2 (Parks et al., 2020). Only genomes with greater than 97 % completeness and less than 3 % contamination were retained. The average nucleotide identity (ANI) among the selected genomes was at least 95 %. Strain source information is provided in Supplementary Table S1.

Genome annotation was performed using Prokka (v1.14.5) to predict protein-coding sequences, rRNAs, and tRNAs (Seemann, 2014). Functional classification of annotated genes was assigned based on the COG and KEGG databases using the eggNOG-mapper tool (http://eggnogdb.embl.de/#/app/home) integrated with the eggNOG orthology framework. Carbohydrate-active enzymes (CAZymes) were identified via the dbCAN2 meta server, with hidden Markov model (HMM)-based searches conducted using HMMER (v3.3.2) (Zhang et al., 2018). The alignment outputs were processed with the hmmscan-parser.sh script provided in the dbCAN toolkit, and only the highest-scoring hit per query was retained for downstream analysis (Yin et al., 2012).

Comparative pan-genome analysis, including delineation of core and accessory gene sets, was conducted using the Roary pipeline (v3.11.2) with a BLASTP sequence identity threshold of 90 % (Page et al., 2015). Host-associated gene enrichment was further assessed using Scoary2 (v2.0.3) (Roder et al., 2024), applying presence/absence matrices from Roary to identify genes significantly associated with poultry- or mammalian-derived strains.

### Probiotic characterization of *L. agilis* 2-2

#### In silico safety assessment and secondary metabolite analysis of L. agilis

The safety profiles of the strains were assessed *in silico*. Antimicrobial resistance genes were identified using ResFinder (https://cge.cbs.dtu.dk/services/ResFinder/), and virulence factors were predicted by BLASTP alignment against the Virulence Factor Database (VFDB) (http://www.mgc.ac.cn/VFs/main.htm) with ≥70 % sequence identity and coverage considered positive. Pathogenic potential was assessed using PathogenFinder 1.1 with default settings (Cosentino et al., 2013). Secondary metabolite biosynthesis was analyzed with antiSMASH (https://antismash.secondarymetabolites.org) across 40 L*. agilis* genomes to identify gene clusters involved in bioactive compound production

#### Short-chain fatty acids (SCFAs) and lactic acid production by *L. agilis* 2-2

SCFA levels were quantified as described (Jacobson et al., 2018) with minor modifications. Briefly, 500 μL of cell-free supernatant was mixed with 30 μL of 0.1 M H₂SO₄, 1 μL 2-methylbutyric acid (internal standard), and 1 mL diethyl ether, followed by centrifugation at 10,000 × g for 15 min at 4 °C. The supernatant was transferred to a tube containing 250 mg sodium carbonate, mixed, and centrifuged to neutralize acidity. The upper phase was filtered (0.22 μm, Millipore) and 1 μL was analyzed on an Agilent 7890/5975 GC-MS with a VF-WAXms column (30 *m* × 0.25 mm, 0.25 μm). Helium was used at 1 mL/min, injector 260°C; the oven was programmed from 80°C to 120°C at 40°C/min, then to 200°C at 10°C/min and held 2 min (total 15 min). Mass spectrometry used electron ionization (70 eV), m/z 30–300, solvent delay 2.5 min. SCFAs were quantified with external calibration standards. Lactic acid levels in supernatants at 24, 36, 48, and 60 h were also measured by HPLC.

#### Acid and bile tolerance of *L. agilis* 2-2

The acid and bile tolerance of *L. agilis* 2-2 was evaluated using modified protocols (Mulaw et al., 2019; Roldán-Pérez et al., 2023). Bacterial suspensions (approximately 1 × 10⁹ CFU/mL) were prepared in MRS broth and incubated at 37°C under two stress conditions. The first condition involved incubation in MRS adjusted to pH 1.5 or 3.0 for 1–3 h to simulate gastric acidity. The second condition involved incubation in MRS supplemented with 0.3 % or 0.7 % (w/v) bile salts for 4 h to mimic intestinal bile exposure. Unmodified MRS broth served as the control. Viability was assessed by plating serial dilutions on MRS agar, and survival rates were expressed as percentages relative to the initial viable counts.

$$\text{Survival}\;\text{rate}=\left(\frac{\text{Final}\;\log\text{CFU}/\text{mL}}{\text{Initial}\;\log\text{CFU}/\text{mL}}\right)\times 100\%$$

#### Cell surface properties and aggregation capacity of *L. agilis* 2-2

Cell surface hydrophobicity of *L. agilis* 2-2 was assessed using a modified microbial adhesion to hydrocarbons (MATH) assay. Bacterial suspensions were standardized to an optical density at 600 nm (OD₆₀₀) of 0.60 ± 0.05 in phosphate-buffered saline (PBS). An aliquot of 4 mL of the suspension was mixed with 0.8 mL of xylene, vortexed, and incubated for phase separation. The OD₆₀₀ of the aqueous phase (A₁) was measured, and hydrophobicity was calculated as:

$$\text{Hydrophobicity}\;=\left(1-\frac{A_{1}}{A_{0}}\right)\times \;100\%$$

where *A₀* is the initial OD₆₀₀ of the bacterial suspension and *A₁* is the OD₆₀₀ of the aqueous phase after mixing with xylene.

Auto-aggregation was evaluated by incubating standardized bacterial suspensions (OD₆₀₀ = 0.60 ± 0.05) at 37°C for up to 24 h, recording OD₆₀₀ at 2, 4, 6, 12, and 24 h. The percentage of auto-aggregation was calculated as:

$$\text{Auto}-\text{aggregation}\;=\left(1-\frac{A_{t}}{A_{0}}\right)\times \;100\%$$

where *A₀* represents the initial OD₆₀₀ of the bacterial suspension and *Aₜ* is the OD₆₀₀ at the indicated time point.

Co-aggregation with pathogens was assessed by mixing equal volumes of *L. agilis* 2-2 and pathogen cultures, followed by incubation at 37°C. Samples were collected at 2, 4, 6, 12, and 24 h, and the absorbance of the mixtures (A_mix_) was measured at 600 nm. Control measurements were obtained from individual suspensions of *L. agilis* (A_LAB_) and the pathogen (A_pathogen_). Co-aggregation rates were calculated using the following formula:

$$\text{Co}-\text{aggregation}\;\text{rate}=\left[1-\frac{A_{mix}}{\left(A_{LAB}+A_{pathogen}\right)/2}\right]\times \;100\%$$

#### Evaluation of antibacterial activity of *L. agilis* 2-2 against pathogens

The antibacterial activity of *L. agilis* 2-2 was evaluated using an Oxford cup assay with minor modifications. The strain was cultured in de MRS broth (AOBOX Biotechnology, China) at 37°C for 24 h under anaerobic conditions, and the cell-free supernatant (CFS) was obtained by centrifugation (7,104 × *g*, 8 min, 4°C; Sigma, Germany) and filtration through 0.22 μm membranes. Indicator pathogens (*Escherichia coli* O78:K80 CICC 10421, *Pseudomonas aeruginosa* PAO1, *Acinetobacter baumannii* ATCC19606^T^, *Staphylococcus aureus* 25993, *Listeria monocytogenes* ATCC 15313^T^ were provided by the Microbiology Laboratory of Northwest University) were cultured overnight in Luria–Bertani (LB) broth (Solarbio, China), adjusted to 1 × 10⁷ CFU/mL (0.5 McFarland standard), and spread (100 μL) onto LB semisolid agar. Oxford cups (8 mm) were placed on plates and filled with 200 μL of CFS; sterile water served as the negative control. Plates were incubated at 37°C for 24 h, and inhibition zones were measured with a Vernier caliper. All assays were performed in triplicate.

#### Evaluation of antioxidant activities of *L. agilis* 2-2

The antioxidant potential of *L. agilis* 2-2 was evaluated using DPPH, hydroxyl radical, and ABTS⁺ radical scavenging assays. For the DPPH assay, bacterial cells were cultured in MRS broth at 37°C for 18 h, harvested, washed, and resuspended in PBS (1 × 10⁹ CFU/mL). A 1.0 mL aliquot of the suspension was mixed with 1.0 mL of 0.02 mM DPPH solution in ethanol, incubated in the dark at 25°C for 30 min, and centrifuged (Kao and Chen, 2006). The absorbance of the supernatant at 517 nm was measured, and scavenging activity was calculated as:

$$\text{Scavenging}\;\text{activity}\;=\left[1-\frac{\left(A_{\text{sample}}-A_{\text{blank}}\right)}{A_{\text{control}}}\right]\times \;100\%$$

where *A*_sample_, *A*_blank_, and *A*_control_ represent the absorbance values of the sample, blank, and control groups, respectively.

Hydroxyl radical scavenging activity was assessed by incubating 0.5 mL of bacterial suspension (1 × 10⁹ CFU/mL) with PBS, 1,10-phenanthroline, FeSO₄, and H₂O₂ at 37°C for 60 min, followed by centrifugation. Absorbance of the supernatant was recorded at 536 nm (Li et al., 2013).

ABTS⁺ radical scavenging was performed by generating ABTS⁺ radicals from ABTS and potassium persulfate, diluting to an absorbance of 1.10 ± 0.02 at 734 nm, and mixing 200 μL of the working solution with 50 μL of bacterial suspension or cell-free supernatant. After 5 min incubation in the dark at room temperature, absorbance at 734 nm was measured (Tsai et al., 2011).

### Statistical analysis

Statistical analyses were conducted using SPSS software (version 22.0) and Python (SciPy package). All experiments were performed in triplicate, and data are presented as mean ± standard error (SE). For pairwise comparisons between two groups, Welch’s *t*-test was applied. Graphical visualizations were generated using R software (v4.2.2) with the ggplot2, reshape2, and tidyverse packages, as well as Python’s Matplotlib library.

## Results

### Genomic characterization and phylogenetic identification of strain 2-2

**Fig. 1:**
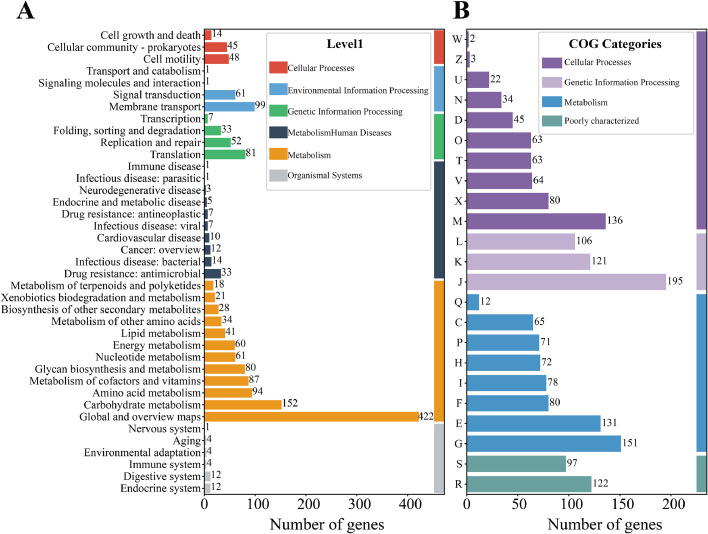
**Functional annotation of the *L. agilis* 2-2 genome.** (A) KEGG functional classification of coding sequences, showing gene enrichment in carbohydrate metabolism, amino acid metabolism, membrane transport, and stress response pathways. (B) COG category distribution of predicted proteins, highlighting dominance of metabolic functions, genetic information processing, and cellular processes, with a substantial proportion of poorly characterized genes.

To explore the genetic underpinning for probiotic potential and host adaptation, we sequenced and analyzed the genome of chicken-derived *L. agilis* strain 2-2. The draft genome of strain 2-2 consists of 73 scaffolds totaling 2.04 Mb, encoding 1,924 protein-coding genes, 9 rRNAs, 87 tRNAs, and 1 tmRNA (Figure S1). KEGG annotation assigned 1,807 genes to functional pathways, with enrichment in carbohydrate, amino acid, and energy metabolism, membrane transport, and signal transduction, indicating metabolic versatility and environmental adaptability (Fig. 1A); COG analysis grouped 1,981 coding sequences into 23 categories, dominated by translation, carbohydrate metabolism, and amino acid metabolism, while 1,219 unclassified genes suggested novel or strain-specific functions (Fig. 1B).

**Fig. 2:**
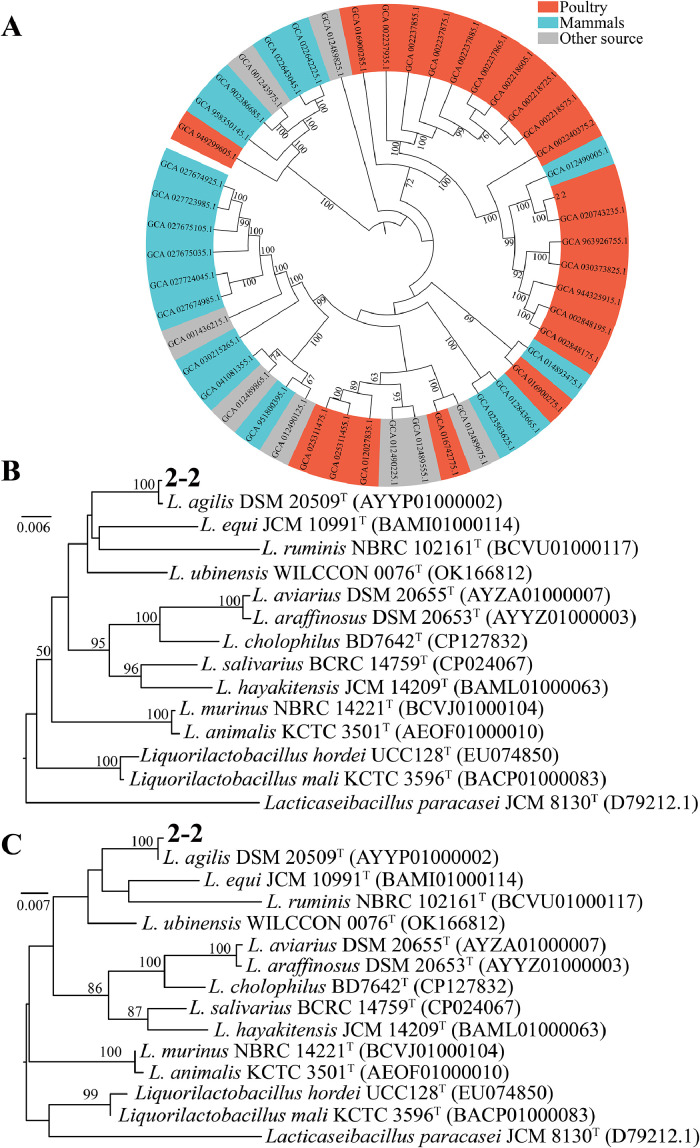
**Phylogenetic analysis of 2-2 and related strains.** (A) Genome-based phylogenetic tree showing host-associated clustering (chicken strains in red, mammalian strains in blue, others in gray), (B) Neighbor-joining (NJ) and (C) maximum-likelihood (ML) trees based on 16S rRNA gene sequences confirm the close relationship of strain 2-2 with *L. agilis* DSM 20509^T^. Bootstrap values >50 % are shown (1,000 replicates).

**Fig. 3:**
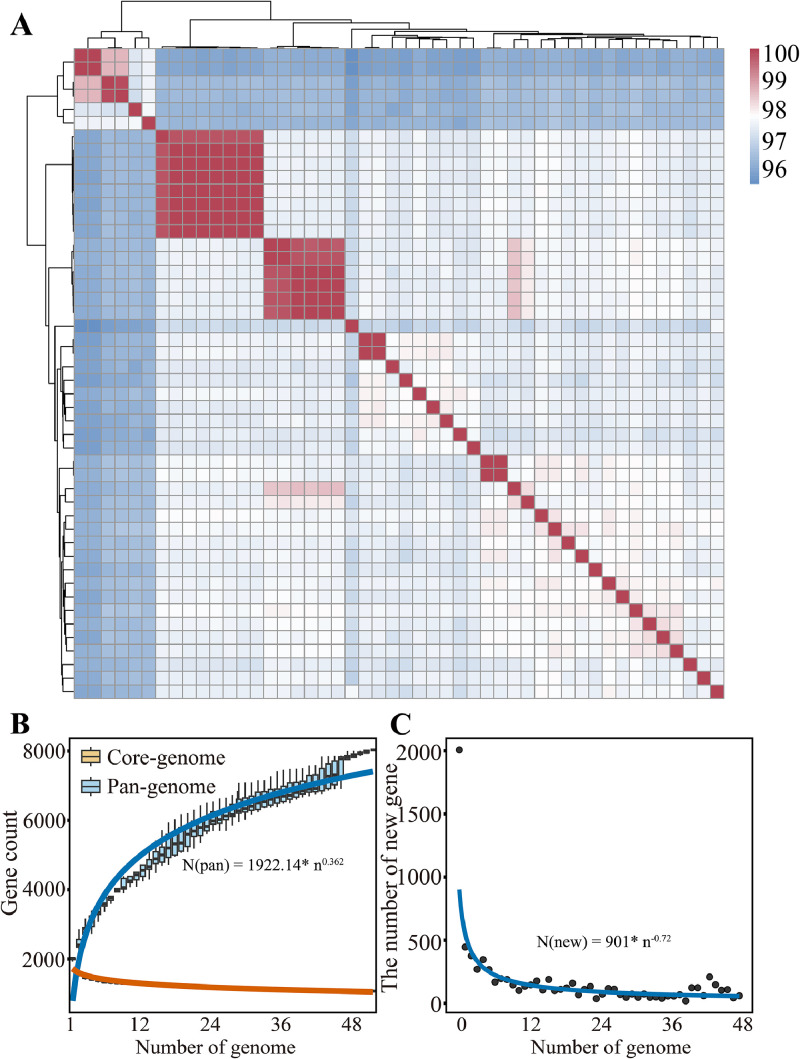
**Genomic similarity and pan-genome analysis of *L. agilis*.** (A) Heatmap of average nucleotide identity (ANI) values among *L. agilis* 2-2 and 47 other *L. agilis* genomes, with hierarchical clustering illustrating genomic relatedness, (B) Gene accumulation curves for the pan-genome (blue) and core genome (orange) derived from 48 L*. agilis* genomes, (C) Gene accumulation curve illustrating the number of new genes identified with the addition of each genome, indicating that the *L. agilis* pan-genome remains open.

Phylogenetic analysis of the 16S rRNA gene placed strain 2-2 in a monophyletic cluster with *L. agilis* DSM 20509^T^, supported by maximum-likelihood and phylogenomic trees (Figs. 2A–C). Genome-based metrics confirmed its species identity, with ANI values exceeding 98 %, AAI of 98.42 %, and dDDH of 92.20 % relative to DSM 20509^T^, and ANI values above 97.14 % compared to 47 L*. agilis* reference genomes (Fig. 3A).

### Pan-genome structure and host-associated genomic divergence of *L. agilis* 2-2

**Fig. 4:**
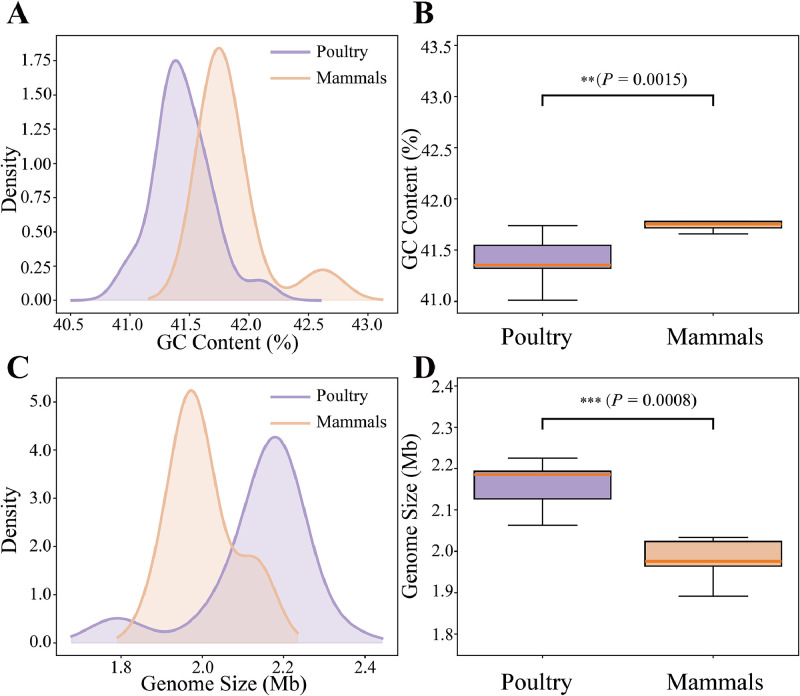
**Host-associated genomic characteristics of *L. agilis* strains.** (A–B) Distribution and boxplot comparison of GC content between poultry- and mammal-derived strains. Mammalian isolates displayed significantly higher GC content than poultry strains (*P* = 0.0015), indicating host-specific genomic variation, (C–D) Distribution and boxplot analysis of genome size, showing that poultry -derived strains generally possess larger genomes than mammalian strains, with statistical significance confirmed (*P* = 0.0008).

Pan-genome analysis of *L. agilis,* incorporating strain 2-2 and 47 publicly available genomes, revealed the impact of host origin on genomic diversity and functional specialization. The analysis identified a total of 8,032 genes, including 1,073 core genes, with a power-law coefficient of 0.362, indicative of an open and expandable pan-genome (Fig. 3B–C). Comparative genomics highlighted clear host-associated adaptations, as poultry-derived strains possessed smaller genomes (1.77–2.34 Mb) with lower GC content (41.0 %–42.1 %), while mammalian strains exhibited larger genomes (1.89–2.96 Mb) and higher GC content (40.1 %–42.6 %) (Fig. 4A–D).

**Fig. 5:**
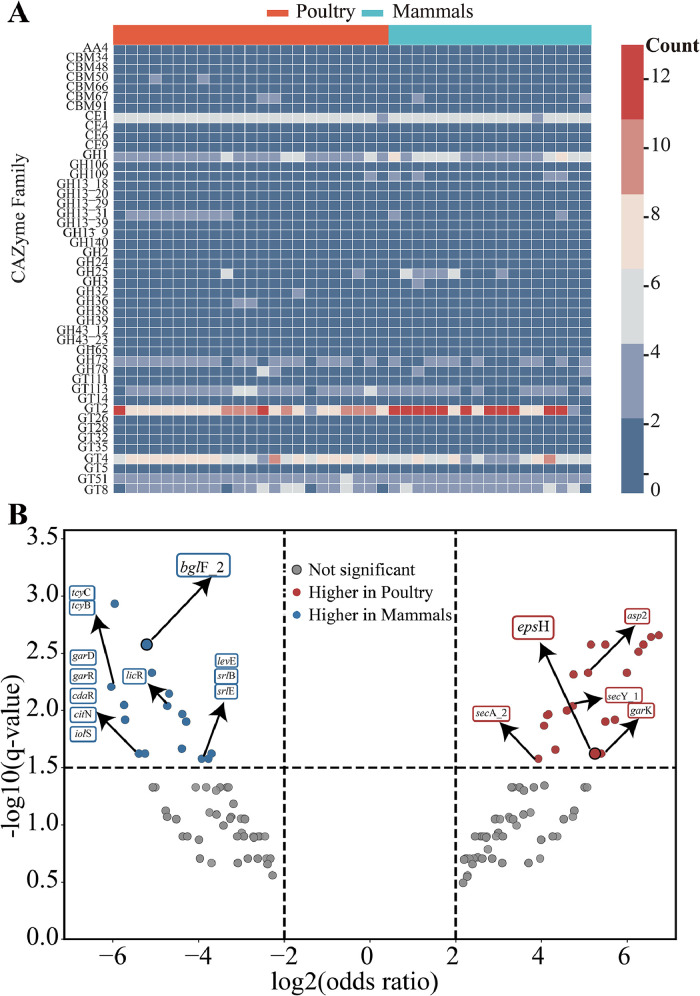
**Comparative analysis of CAZymes and host-associated gene enrichment in *L. agilis*.** (A) Heatmap showing the distribution of CAZyme families across poultry- and mammalian-derived genomes (red, poultry; blue, mammals). (B) Volcano plot of genes differentially enriched between poultry and mammalian strains based on Scoary analysis, with poultry-enriched genes in red (*eps*H), mammalian-enriched genes in blue (*bgl*F_2), and non-significant genes in gray.

Analysis of carbohydrate-active enzymes (CAZymes) showed that the glycosyltransferase family GT2 was significantly enriched in mammalian-derived *L. agilis* strains (Fig. 5A), whereas its abundance was markedly lower in poultry isolates. Scoary2-based genome-wide association further identified two host-biased carbohydrate-related genes, with the exopolysaccharide biosynthesis gene *eps*H enriched in poultry-derived strains and the β-glucoside PTS transporter gene *bgl*F_2 enriched in mammalian-derived strains (Fig. 5B; Table S2). These findings demonstrate clear host-driven divergence in carbohydrate-related functional traits of *L. agilis*.

### *In-vitro* probiotic potential assessment of *L. agilis* 2-2

#### Genomic prediction of safety and secondary metabolite potential in *L. agilis* 2-2

To further evaluate the safety and functional potential of *L. agilis* 2-2, we examined its genome for antimicrobial resistance, virulence, and secondary metabolite genes. Only a single *vanT-*like resistance gene was detected, with low similarity and an incomplete operon, indicating minimal functional significance. Three virulence-associated genes (*has*C, *clp*P, *tuf*A) were also detected, but these are common in commensal lactobacilli and linked to stress tolerance or adhesion rather than pathogenicity. PathogenFinder predicted a very low pathogenicity probability (0.0795), confirming its genomic safety.

**Fig. 6:**
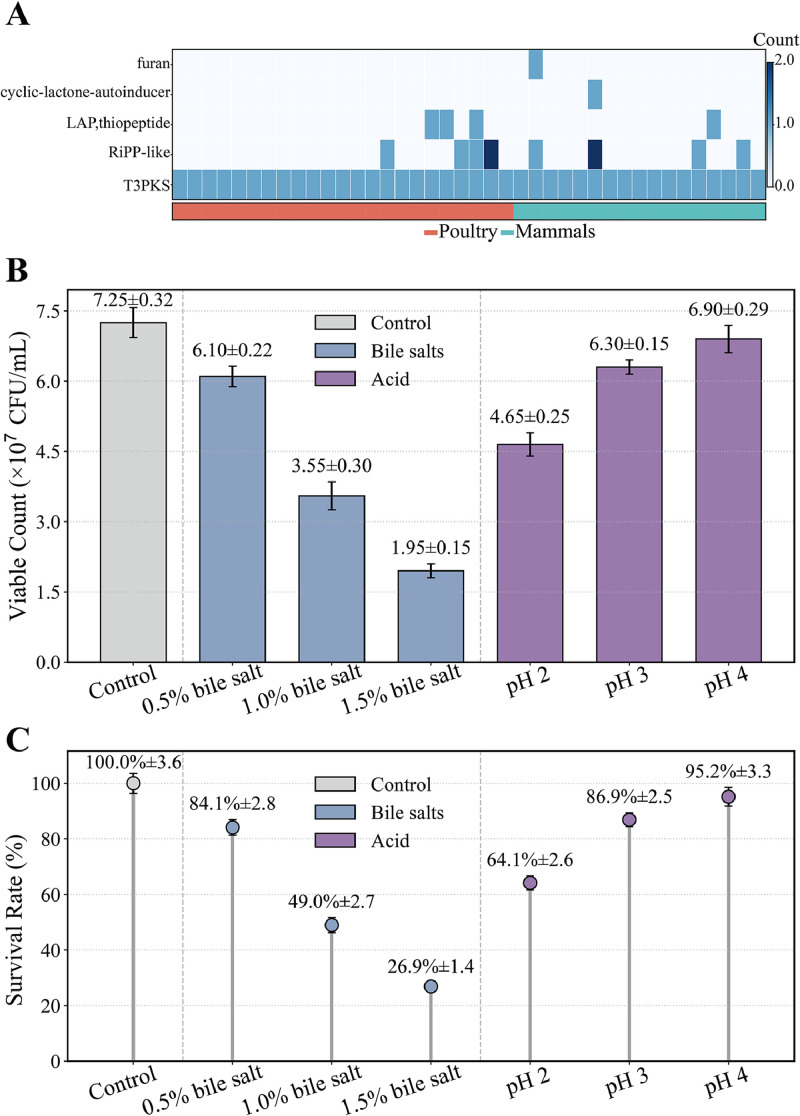
**Secondary metabolites and survival of *L. agilis* 2-2 under simulated gastrointestinal conditions.** (A) Distribution of biosynthetic gene clusters (BGCs) among poultry- and mammalian-derived *L. agilis* genomes. (B) Viable cell counts (×10⁷ CFU/mL) of *L. agilis* 2-2 after exposure to bile salts (0.5 %, 1.0 %, and 1.5 %) and acidic conditions (pH 2, 3, and 4) for the indicated times. (C) Corresponding survival rates calculated as the ratio of viable cells after treatment to the initial cell count. Data are presented as mean ± standard deviation (SD) from three independent experiments.

AntiSMASH analysis identified five biosynthetic gene cluster types across *L. agilis* (Fig. 6A), with T3PKS universally conserved and other clusters (RiPP-like, LAP/thiopeptide, cyclic-lactone autoinducer, furan) sporadically distributed, showing no major host-associated differences. Strain 2-2 carried the conserved T3PKS cluster, typically linked to antimicrobial and antioxidant activities.

In addition, genome annotation further revealed several probiotic-related gene clusters involved in acid and bile tolerance, antioxidant defense, antimicrobial activity, adhesion, and SCFAs metabolism (Supplementary Figure S2). The atpA–atpH operon and groEL/groES chaperone system support proton extrusion and protein homeostasis under acid and bile stress. Redox-regulating genes (trxA/B, msrA/B, ohrR) form an integrated antioxidant network that mitigates oxidative damage. Adhesion-associated loci (lmrB, prsA, lspA) enhance surface hydrophobicity, aggregation, and mucosal attachment, promoting intestinal persistence. Metabolic genes (ldhA, ackA–pta, pdhABC) mediate lactic acid and SCFAs synthesis, contributing to gut acidification and pathogen inhibition. These findings indicate that *L. agilis* 2-2 lacks clinically relevant resistance or virulence determinants and harbors only non-pathogenic biosynthetic gene clusters (BGCs), reinforcing both its safety and functional potential.

#### Evaluation of gastrointestinal adaptation, metabolite production, and adhesion traits in *L. agilis* 2-2

The ability to endure acidic and bile conditions, generate metabolites that modulate the gut environment, and adhere to intestinal surfaces supports the ecological fitness of a probiotic. *L. agilis 2-2* showed substantial tolerance to bile salts and acidic conditions, reflecting its potential to endure the avian gut environment. The strain-maintained survival rates of 84.14 %, 48.97 %, and 26.90 % under 0.5 %, 1.0 %, and 1.5 % bile salts, respectively, and survived at pH 4.0 (95.17 %), pH 3.0 (86.90 %), and pH 2.0 (64.14 %) (Fig. 6B–C). These findings demonstrate that *L. agilis* 2-2 can effectively endure the bile and acidic stress typical of the avian gastrointestinal tract, suggesting its potential for successful gastrointestinal transit and colonization in poultry.

**Fig. 7:**
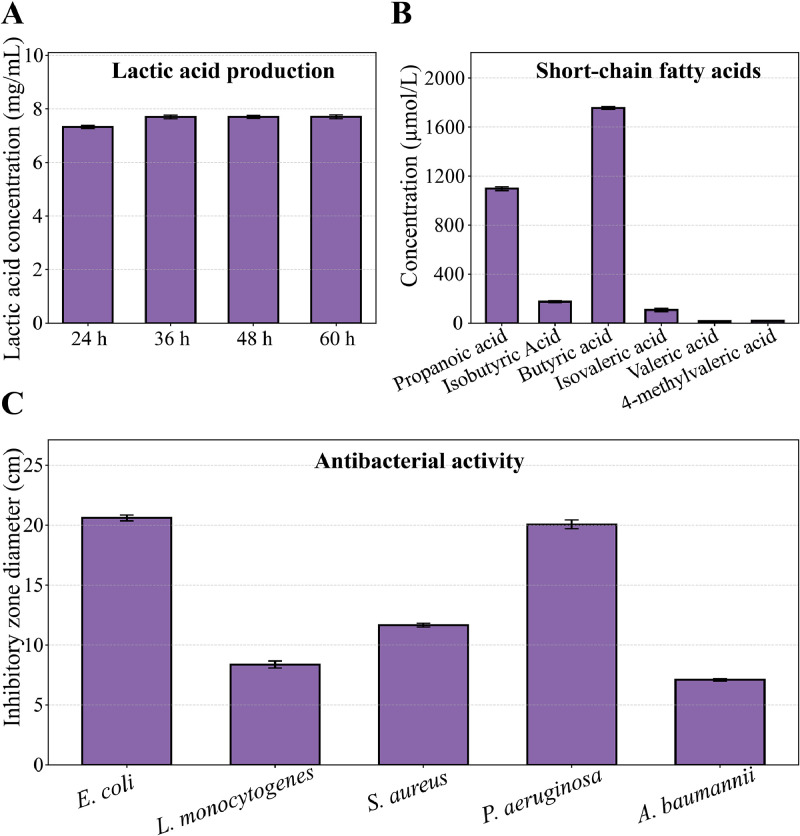
**Antibacterial activity, lactic acid production and SCFAs production of *L. agilis* 2-2.** (A) Lactic acid production by strain 2-2 over time during fermentation. (B) SCFAs profile of strain 2-2 fermentation supernatant, dominated by butyrate and propionate with moderate isobutyrate and isovalerate, and trace valerate and 4-methylvalerate. (C) Antibacterial activity of strain 2-2 against poultry-associated pathogens.

Lactic acid and SCFAs are critical metabolites for maintaining gut homeostasis, inhibiting pathogens, and supporting intestinal barrier function. *L. agilis* 2-2 gradually accumulated lactic acid during fermentation, reaching 7.32 ± 0.06 mg/mL at 24 h and 7.69 ± 0.07 mg/mL at 36 h, and remaining stable at 7.60–7.70 mg/mL at 48–60 h (Fig. 7A). The strain also generated diverse short-chain fatty acids (SCFAs), with butyrate predominating (1,767.2 ± 4.2 μg/mL), followed by propionate (1,097.0 ± 22.7 μg/mL), and moderate levels of isobutyrate (174.4 ± 9.6 μg/mL) and isovalerate (107.3 ± 23.2 μg/mL) (Fig. 7B). These metabolites contribute to intestinal barrier integrity, pathogen inhibition, and gut homeostasis.

**Fig. 8:**
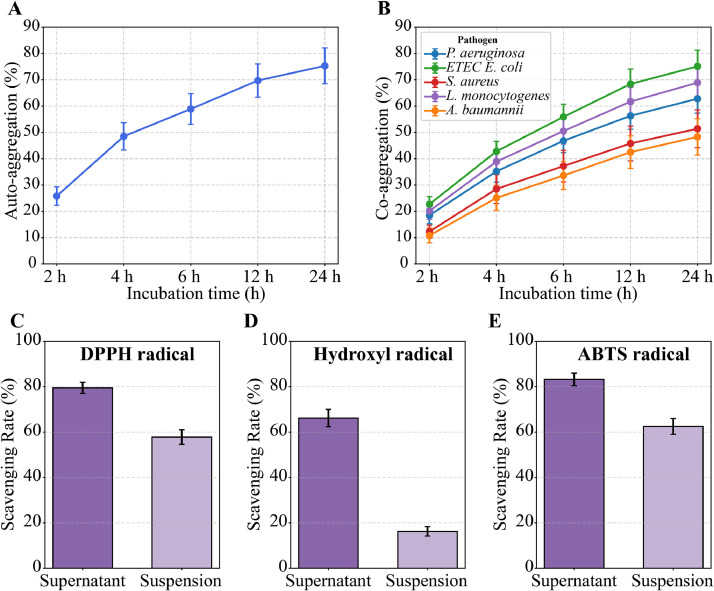
**Auto-aggregation, co-aggregation, and antioxidant activity of *L. agilis* 2-2.** (A) Auto-aggregation capacity of *L. agilis* 2-2 during 24 h incubation, showing a progressive increase in aggregation percentage over time. (B) Time-dependent co-aggregation of *L. agilis* 2-2 with common poultry pathogens, including *E. coli* (ETEC), *P. aeruginosa, S. aureus, L. monocytogenes*, and *A. baumannii*. (C–E) Antioxidant activity of *L. agilis* 2-2 determined by DPPH (C), hydroxyl (D), and ABTS (E) radical scavenging assays. Data represent mean ± standard deviation from three independent experiments.

In addition*, L. agilis* 2-2 displayed strong aggregation-related traits that facilitate gut colonization. Auto-aggregation increased from 25 % at 2 h to nearly 75 % at 24 h (Fig. 8A), and cell surface hydrophobicity was high (55.3 ± 4.8 %), supporting adhesion to epithelial surfaces. Time-dependent co-aggregation with pathogens was observed, strongest with *E. coli*, followed by *L. monocytogenes* and *P. aeruginosa*, with moderate interaction with *S. aureus* and *A. baumannii* (Fig. 8B). Collectively, these findings indicate that *L. agilis* 2-2 combines stress tolerance, metabolite production, and adhesion capacity to effectively colonize the gut and compete against pathogens.

#### Antimicrobial and antioxidant activities of *L. agilis* 2-2

Antimicrobial and antioxidant activities contribute to gut health by limiting pathogen colonization, reducing oxidative stress, and supporting intestinal barrier integrity. *L. agilis* 2-2 exhibited broad-spectrum antimicrobial activity against five poultry-associated pathogens (Fig. 7C). The strongest inhibition was observed against *E. coli* (20.60 ± 0.63 mm) and *P. aeruginosa* (20.13 ± 0.42 mm), major agents of enteric infections in poultry. Moderate inhibition occurred against *S. aureus* (11.66 ± 0.28 mm) and *L. monocytogenes* (8.04 ± 0.10 mm), relevant to foodborne diseases, while the weakest effect was observed against *A. baumannii* (7.09 ± 0.16 mm). These results indicate pronounced antimicrobial activity, particularly against Gram-negative bacteria, supporting the strain’s potential to prevent pathogen colonization in the poultry gut.

*L. agilis* 2-2 exhibited notable antioxidant activity, as assessed by DPPH, hydroxyl, and ABTS⁺ radical scavenging assays (Fig. 8C–E). Both intact cells and the cell-free supernatant showed free radical scavenging capacity, with the supernatant consistently more effective. The supernatant achieved scavenging rates of 79.5 ± 2.5 %, 66.2 ± 3.8 %, and 83.2 ± 2.8 % against DPPH, hydroxyl, and ABTS⁺ radicals, respectively, compared with 57.8 ± 3.2 %, 16.3 ± 2.1 %, and 62.5 ± 3.5 % for intact cells. ABTS⁺ radicals were neutralized most efficiently, followed by hydroxyl and DPPH radicals. This suggests that antioxidant metabolites released into the medium contribute to protection against oxidative stress. Together, the broad-spectrum antimicrobial activity and antioxidant capacity highlight *L. agilis* 2-2 as a promising probiotic capable of enhancing intestinal homeostasis and poultry gut health.

## Discussion

### Genomic profile of *L. agilis* 2-2 reveals broad functional versatility and safety

Probiotic microorganisms have drawn increasing scientific and clinical attention for their ability to modulate gut microbiota, strengthen intestinal barriers, and regulate immune responses (La Fata et al., 2018). Among them, lactic acid bacteria (LAB) are widely recognized for their safety and multifunctionality in both clinical and agricultural contexts. Host-derived isolates often exhibit enhanced ecological compatibility and colonization efficiency (Li and Gänzle, 2020). In this study, *L. agilis* 2-2, isolated from the intestine of a healthy chicken, displayed a genomic feature consistent with strong metabolic adaptability and environmental resilience.

Functional enrichment in carbohydrate, amino acid, and energy metabolism pathways reveals a metabolically versatile profile in L. agilis 2-2, consistent with its probiotic adaptability (Siezen et al., 2011; Luo et al., 2021). Abundant genes related to membrane transport and signal transduction further support efficient nutrient uptake and environmental responsiveness (Duca et al., 2021; Wan et al., 2023). COG analysis emphasized functions associated with translation, amino acid metabolism, and cell envelope biogenesis, reflecting stress resilience and cellular integrity (Mitchell et al., 2019; Schöp et al., 2023). Notably, more than 1,200 unclassified genes may encode previously unrecognized mechanisms contributing to probiotic functionality.

Genomic safety assessment revealed only a single, incomplete *van*T-like antimicrobial resistance gene with low identity, lacking a functional operon or mobile elements, suggesting negligible transfer potential. Similarly, the annotated “virulence-related” genes (*has*C, *clp*P, *tuf*A) represent common housekeeping functions involved in polysaccharide synthesis, stress response, and adhesion rather than pathogenicity. This was corroborated by a very low PathogenFinder probability (<0.08) (Schmid et al., 2015; Sun et al., 2023; Du et al., 2023). Overall, the genomic safety and metabolic characters of *L. agilis* 2-2 closely resemble established poultry probiotics such as *L. salivarius* and *L. johnsonii* (Jiang et al., 2023; Johnson et al., 2023), supporting its candidacy as a host-adapted probiotic with low safety risk.

### Host-driven evolutionary divergence and pan-genome dynamics of *L. agilis*

Pan-genome analysis of *L. agilis* revealed an open structure (power-law coefficient 0.362) comprising 8,032 gene clusters, indicative of high genomic plasticity and active gene acquisition. Phylogenetic analysis showed that *L. agilis* did not cluster strictly by host origin, implying functional diversification driven by ecological adaptation rather than lineage (Schuster et al., 2020; You et al., 2023). This pattern is consistent with *Lactiplantibacillus plantarum,* which inhabits multiple niches and exhibits extensive genotypic diversity (Zhang et al., 2021; Huang et al., 2021).

Population genomic analysis revealed distinct adaptive trajectories between poultry- and mammal-derived *L. agilis* strains. Poultry isolates, including strain 2-2, possessed larger genomes, reflecting expanded metabolic and adaptive capacities, whereas mammalian isolates exhibited streamlined genomes characteristic of niche specialization (Wang et al., 2023). The avian gut’s fluctuating nutritional features and high microbial diversity likely promote horizontal gene transfer and genome expansion (de Sousa et al., 2023), while mammalian hosts exert more stable, lineage-specific selection favoring genomic reduction (Siozios et al., 2024). Lower GC content in poultry strains may reduce nucleotide synthesis costs, whereas higher GC in mammalian strains supports refined transcriptional regulation (Zrimec et al., 2022).

Comparative analysis showed that poultry and mammalian isolates shared a conserved CAZyme repertoire, with host-specific differences. Glycosyltransferase GT2 was enriched in mammalian strains, suggesting enhanced peptidoglycan and polysaccharide biosynthesis (Stone et al., 2010). In contrast, the capsule biosynthesis gene *eps*H*,* prevalent in poultry strains, may promote persistence in the avian gut (Jiang et al., 2025). Genome-wide association further identified *bgl*F_2, a β-glucoside phosphotransferase enriched in mammalian isolates, potentially facilitating host glycan utilization (Braza et al., 2020). These findings indicate that *L. agilis* maintains a stable core metabolism while exhibiting host-specific genomic adaptations (Lv et al., 2024).

### Functional mechanisms underlying the probiotic potential of *L. agilis 2-2*

The functional characteristics of *L. agilis* 2-2 integrate metabolic activity, surface adhesion, and pathogen inhibition into a coherent probiotic profile. The strain produced substantial lactic acid during fermentation, which can lower pH and potentiate antimicrobial effects, particularly against *E. coli* and *P. aeruginosa*, major poultry enteropathogens. Additional inhibition of *S. aureus* and *L. monocytogenes* confirmed its broad-spectrum antagonistic capacity.

Beyond organic acids, *L. agilis* 2-2 generated diverse short-chain fatty acids (SCFAs), dominated by butyrate and propionate, which serve critical roles in intestinal barrier integrity, energy metabolism, and immune modulation (Yao et al., 2022; Liu et al., 2021). The detection of branched-chain SCFAs such as isobutyrate and isovalerate further indicates metabolic flexibility and cross-feeding potential within gut consortia (Tsukuda et al., 2021).

Surface physicochemical properties reinforced these metabolic traits (Boonaert and Rouxhet., 2000). *L. agilis* 2-2 exhibited high hydrophobicity and strong auto-aggregation, facilitating mucosal adhesion and persistence, while significant co-aggregation with *E. coli* and *L. monocytogenes* suggests a competitive exclusion mechanism (Ahmed, Ahmed and Khan, 2018). Genes associated with acid and bile tolerance, antioxidant defense, adhesion, and antimicrobial production were also identified, supporting a genomic foundation for its probiotic phenotype. Compared with the poultry strain *L. agilis* 1003 (Yin et al., 2024) and the canine-derived strain *L. agilis* L44 (Liu et al., 2024), strain 2-2 showed equal or higher survival under bile and acid stress, with auto-aggregation and cell surface hydrophobicity within their reported ranges. It also displayed a broader antimicrobial spectrum against poultry pathogens and robust antioxidant activity, suggesting that 2-2 may match or even surpass previously described *L. agilis* strains in key probiotic traits. Together, these results indicate that *L. agilis* 2-2 integrates acidification, SCFAs production, adhesion, and pathogen exclusion to support intestinal homeostasis and host health. Future studies should evaluate *L. agilis* 2-2 in relevant poultry models to assess its physiological effects, colonization efficiency, and probiotic efficacy, and to determine whether the host-specific genomic features observed here translate into measurable health benefits under practical production and management conditions.

## Conclusion

This study presents the first comprehensive pan-genomic and functional characterization of *L. agilis*, focusing on the chicken-derived strain 2-2. Comparative genomics revealed clear host-associated differences, with poultry *L. agilis* isolates carrying larger genomes enriched in glycoside hydrolases and mammalian strains enriched in glycosyltransferases, reflecting ecological and dietary adaptation. Strain 2-2 displayed multiple probiotic traits, including acid and bile tolerance, strong aggregation, broad-spectrum antimicrobial activity, antioxidant capacity, and substantial production of short-chain fatty acids, particularly butyrate and propionate. Together, these genomic and phenotypic features establish *L. agilis* 2-2 as a metabolically versatile, safe, and host-adapted probiotic. These findings provide new insights into *L. agilis* evolution and support its potential application in enhancing poultry gut health.

## CRediT authorship contribution statement

**Zhen Zhang:** Writing – original draft, Investigation, Formal analysis, Data curation. **Yang Lv:** Writing – review & editing, Investigation. **Zisheng Guo:** Visualization, Validation, Supervision, Formal analysis. **Lei Liu:** Methodology, Investigation. **Xiaohui Chen:** Writing – review & editing, Data curation. **Wenjing Han:** Writing – review & editing. **Jinshuo Wei:** Writing – review & editing. **Songtao Guo:** Supervision, Funding acquisition, Conceptualization. **Yanmei Sun:** Supervision, Funding acquisition, Conceptualization. **Shiwei Wang:** Supervision, Resources, Project administration, Funding acquisition, Conceptualization.

## Disclosures

The authors declare that they have no known competing financial interests or personal relationships that could have appeared to influence the work reported in this paper.

## Appendix Supplementary materials

Image, application 1

## Footnotes

Supplementary material associated with this article can be found, in the online version, at doi:10.1016/j.psj.2025.106274.
