# Supplementary material for: Host-adapted probiotic potential of Ligilactobacillus agilis 2-2 revealed by comparative genomic and phenotypic analyses
Source: Poult Sci. 2025 Dec 12;105(2):106274. doi: 10.1016/j.psj.2025.106274 (PMC12774754; doi:10.1016/j.psj.2025.106274)
Supplement: Supplementary file 1 [file mmc1.docx]

Host-adapted probiotic potential of *Ligilactobacillus agilis* 2-2 revealed by comparative genomic and phenotypic analyses

Zhen Zhang^a,1^, Yang Lv^b,1^, Zisheng Guo^a^ , Lei Liu^a^, Xiaohui Chen^a,d^, Wenjing Han^a^, Jinshuo Wei^a^, Songtao Guo^c^, Yanmei Sun^a,*^ Shiwei Wang^a,*^

^a^ Key Laboratory of Resource Biology and Biotechnology in Western China, Ministry of Education, Provincial Key Laboratory of Biotechnology, College of Life Sciences, Northwest University, 229 Taibai North Road, Xi’an, Shaanxi 710069, China.

^b^ Institute of Biomedical Research, Henan Academy of Sciences, Zhengzhou 450002, China

^c^ Shaanxi Key Laboratory for Animal Conservation, College of Life Sciences, Northwest University, Xi’an, Shaanxi 710069, China.

^d^ Jian Yang City Product Quality Supervision & Testing Institute, Jianyang, China

**^1^** These authors contributed equally to this work.

* Corresponding author:

**Yanmei Sun:** sun[yanmei@nwu.edu.cn](mailto:yanmeisun@nwu.edu.cn)

**Shiwei Wang:** [wangsw@nwu.edu.cn](mailto:wangsw@nwu.edu.cn)

**Table S1** Genome features and assembly quality statistics of *L. agilis* strains isolated from different hosts.

| Genome ID | Genome size (bp) | Source | GC content (%) | Compl-eteness | Conta-mination |
| --- | --- | --- | --- | --- | --- |
| GCA_002218575.1 | 2187786 | turkeys | 41.34 | 98.69 | 0.52 |
| GCA_002218605.1 | 2188001 | turkeys | 41.32 | 98.69 | 0.52 |
| GCA_002218725.1 | 2188892 | turkeys | 41.33 | 98.69 | 0.52 |
| GCA_002237855.1 | 2185895 | turkeys | 41.36 | 98.69 | 0.52 |
| GCA_002237865.1 | 2189177 | turkeys | 41.34 | 98.69 | 0.52 |
| GCA_002237875.1 | 2195660 | turkeys | 41.35 | 98.69 | 0.52 |
| GCA_002237885.1 | 2191601 | turkeys | 41.32 | 98.69 | 0.52 |
| GCA_002237935.1 | 2210666 | turkeys | 41.35 | 98.69 | 0.52 |
| GCA_002240375.2 | 2205859 | chicken | 41.55 | 98.69 | 0.79 |
| GCA_002848175.1 | 2166308 | chicken | 41.29 | 98.17 | 1.62 |
| GCA_002848195.1 | 2138099 | chicken | 41.32 | 98.69 | 1.62 |
| GCA_012027835.1 | 2090135 | chicken | 41.73 | 98.69 | 0.26 |
| GCA_016742775.1 | 2342030 | chicken | 41.02 | 98.69 | 1.62 |
| GCA_016900275.1 | 2172358 | chicken | 41.53 | 98.69 | 1.31 |
| GCA_016900285.1 | 2197849 | chicken | 41.47 | 98.69 | 0.26 |
| GCA_020743235.1 | 1778556 | chicken | 42.1 | 98.17 | 0.26 |
| GCA_025311455.1 | 2168186 | chicken | 41.54 | 98.69 | 0.52 |
| GCA_025311475.1 | 2162388 | chicken | 41.52 | 98.69 | 0.52 |
| GCA_030373825.1 | 2225272 | chicken | 41.01 | 98.69 | 0.65 |
| GCA_944325915.1 | 2007959 | chicken | 41.65 | 98.17 | 0.54 |
| GCA_949299605.1 | 2115186 | chicken | 41.59 | 98.17 | 0.26 |
| GCA_963926755.1 | 1801818 | chicken | 41.74 | 97.64 | 0.8 |
| 2-2 | 2063074 | chicken | 41.6 | 98.69 | 0.79 |
| GCA_014893475.1 | 2239969 | calf | 41.54 | 98.69 | 0.26 |
| GCA_022642225.1 | 2065732 | calf | 41.81 | 98.69 | 1.05 |
| GCA_022643045.1 | 2059267 | calf | 41.79 | 98.17 | 1.05 |
| GCA_041081355.1 | 2166317 | calf | 41.7 | 98.69 | 0.26 |
| GCA_001243975.1 | 2133538 | human | 41.74 | 98.17 | 1.83 |
| GCA_027674925.1 | 1966540 | human | 41.71 | 98.69 | 0.26 |
| GCA_027674985.1 | 1963711 | human | 41.78 | 98.69 | 0.26 |
| GCA_027675035.1 | 1949555 | human | 41.77 | 98.69 | 0.26 |
| GCA_027675105.1 | 1994370 | human | 41.66 | 98.69 | 0.26 |
| GCA_027723985.1 | 1985125 | human | 41.68 | 98.69 | 0.26 |
| GCA_027724045.1 | 1965638 | human | 41.78 | 98.69 | 0.26 |
| GCA_030215265.1 | 2033448 | human | 41.95 | 98.69 | 0.79 |
| GCA_902386685.1 | 2133538 | human | 41.74 | 98.17 | 1.83 |
| GCA_958350145.1 | 1891329 | human | 42.62 | 98.69 | 0.79 |
| GCA_951800395.1 | 1896567 | mice | 42.11 | 97.47 | 0.79 |
| GCA_012843665.1 | 2204013 | Pig | 41.35 | 98.69 | 0.79 |
| GCA_023563625.1 | 2961932 | Pig | 40.15 | 98.69 | 0.26 |
| GCA_001436215.1 | 2058058 | Sewage | 41.74 | 98.69 | 0.26 |
| GCA_012489555.1 | 2524354 | Unknown | 40.53 | 98.69 | 0.76 |
| GCA_012489675.1 | 2258084 | Unknown | 41.38 | 98.69 | 0.26 |
| GCA_012489825.1 | 2137178 | Unknown | 41.7 | 98.69 | 0.26 |
| GCA_012489865.1 | 2253382 | Unknown | 41.35 | 98.69 | 1.05 |
| GCA_012490005.1 | 2113821 | Unknown | 41.66 | 98.69 | 1.05 |
| GCA_012490125.1 | 2033535 | Unknown | 41.69 | 98.69 | 0.79 |
| GCA_012490225.1 | 2212130 | Unknown | 41.45 | 98.69 | 0.26 |

**
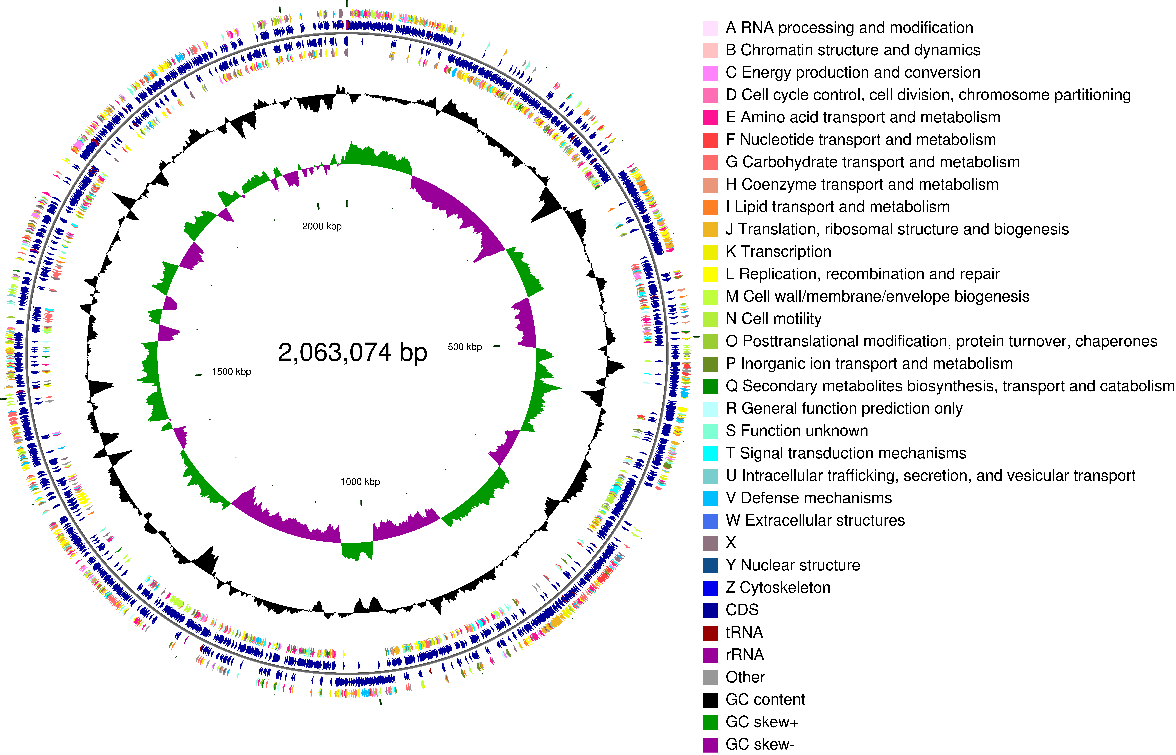
**

**Figure S1. Genome visualization and annotation of 2-2.** The map displays the genomic architecture of strain 2-2 (2,063,074 bp). From the outer to inner rings: predicted coding sequences (CDSs) on the forward and reverse strands colored by COG functional categories, tRNA and rRNA genes, and other annotated features. The innermost rings show GC content (black) and GC skew (green/purple).

**Table S2** Top 100 significantly differentially enriched genes between chicken- and mammalian-derived *L. agilis* strains identified by Scoary analysis.

| Gene | Odds_ratio | Benjamini_H_p | Enriched_in |
| --- | --- | --- | --- |
| group_1049 | inf | 0.0022 | Poultry high |
| group_1790 | inf | 0.0022 | Poultry high |
| group_38 | 71.5 | 0.0027 | Poultry high |
| group_790 | 49 | 0.0027 | Poultry high |
| group_1725 | inf | 0.0027 | Poultry high |
| group_1726 | inf | 0.0027 | Poultry high |
| group_2292 | inf | 0.0027 | Poultry high |
| group_2293 | inf | 0.0027 | Poultry high |
| group_2294 | inf | 0.0027 | Poultry high |
| group_136 | inf | 0.0031 | Poultry high |
| group_809 | inf | 0.0047 | Poultry high |
| *asp*2 | 52.8 | 0.0047 | Poultry high |
| group_1894 | 35.625 | 0.0048 | Poultry high |
| *sec*Y_1 | 40.333 | 0.0091 | Poultry high |
| group_2330 | 36.571 | 0.0099 | Poultry high |
| group_3109 | 36.571 | 0.0099 | Poultry high |
| group_3110 | 36.571 | 0.0099 | Poultry high |
| group_438 | 22.167 | 0.0108 | Poultry high |
| group_334 | 21.667 | 0.0110 | Poultry high |
| *yne*A | inf | 0.0120 | Poultry high |
| group_2231 | inf | 0.0125 | Poultry high |
| group_270 | inf | 0.0125 | Poultry high |
| group_365 | 21.25 | 0.0136 | Poultry high |
| *his*C_1 | 30 | 0.0220 | Poultry high |
| *gar*K | inf | 0.024 | Poultry high |
| group_3519 | inf | 0.024 | Poultry high |
| group_3107 | inf | 0.024 | Poultry high |
| group_2331 | inf | 0.024 | Poultry high |
| *eps*H | inf | 0.024 | Poultry high |
| group_569 | inf | 0.024 | Poultry high |
| group_1845 | inf | 0.024 | Poultry high |
| group_1768 | inf | 0.024 | Poultry high |
| *sec*A_2 | 19.25 | 0.026 | Poultry high |
| group_1545 | 24.889 | 0.045 | Poultry high |
| group_1546 | 24.889 | 0.045 | Poultry high |
| group_506 | 0 | 0.0004 | Mammals high |
| group_2424 | 0.010 | 0.001 | Mammals high |
| *bgl*F_2 | 0.017 | 0.002 | Mammals high |
| group_716 | 0.0189 | 0.004 | Mammals high |
| group_1421 | 0 | 0.006 | Mammals high |
| group_2406 | 0 | 0.006 | Mammals high |
| *tcy*B | 0 | 0.006 | Mammals high |
| group_2416 | 0 | 0.006 | Mammals high |
| *tcy*C | 0 | 0.006 | Mammals high |
| group_985 | 0.029 | 0.007 | Mammals high |
| group_1051 | 0 | 0.009 | Mammals high |
| group_2410 | 0 | 0.009 | Mammals high |
| *srl*B | 0.025 | 0.009 | Mammals high |
| *srl*E | 0.025 | 0.009 | Mammals high |
| group_3162 | 0.025 | 0.009 | Mammals high |
| *lic*R | 0.025 | 0.009 | Mammals high |
| group_3165 | 0.025 | 0.009 | Mammals high |
| group_1444 | 0.025 | 0.009 | Mammals high |
| group_1140 | 0.025 | 0.009 | Mammals high |
| group_3160 | 0.025 | 0.009 | Mammals high |
| *vdc*A | 0.037 | 0.011 | Mammals high |
| group_1902 | 0 | 0.012 | Mammals high |
| group_232 | 0 | 0.012 | Mammals high |
| group_722 | 0 | 0.012 | Mammals high |
| *liv*H | 0.040 | 0.013 | Mammals high |
| *bra*C | 0.040 | 0.013 | Mammals high |
| *liv*F | 0.040 | 0.013 | Mammals high |
| group_2361 | 0.040 | 0.013 | Mammals high |
| *lpt*B | 0.040 | 0.013 | Mammals high |
| group_140 | 0.032 | 0.022 | Mammals high |
| group_402 | 0.032 | 0.022 | Mammals high |
| group_600 | 0.032 | 0.022 | Mammals high |
| *gar*D | 0 | 0.024 | Mammals high |
| group_4225 | 0 | 0.024 | Mammals high |
| *cit*N | 0 | 0.024 | Mammals high |
| *gar*R | 0 | 0.024 | Mammals high |
| *gud*D_1 | 0 | 0.024 | Mammals high |
| *dap*A_1 | 0 | 0.024 | Mammals high |
| *iol*S | 0 | 0.024 | Mammals high |
| group_477 | 0 | 0.024 | Mammals high |
| group_2881 | 0 | 0.024 | Mammals high |
| *gud*D_2 | 0 | 0.024 | Mammals high |
| *cda*R | 0 | 0.024 | Mammals high |
| group_4230 | 0 | 0.024 | Mammals high |
| group_254 | 0.065 | 0.024 | Mammals high |
| group_1597 | 0.065 | 0.024 | Mammals high |
| group_1355 | 0 | 0.024 | Mammals high |
| group_1521 | 0.052 | 0.026 | Mammals high |
| *lev*D | 0.058 | 0.026 | Mammals high |
| *lev*E | 0.058 | 0.026 | Mammals high |
| group_5631 | 0 | 0.047 | Mammals high |
| group_5075 | 0 | 0.047 | Mammals high |
| group_5076 | 0 | 0.047 | Mammals high |
| group_5077 | 0 | 0.047 | Mammals high |
| group_5078 | 0 | 0.047 | Mammals high |
| group_338 | 0 | 0.047 | Mammals high |
| group_5079 | 0 | 0.047 | Mammals high |
| group_5566 | 0 | 0.047 | Mammals high |
| group_5567 | 0 | 0.047 | Mammals high |
| group_5610 | 0 | 0.047 | Mammals high |
| group_5611 | 0 | 0.047 | Mammals high |
| group_567 | 0 | 0.047 | Mammals high |
| group_3315 | 0.085 | 0.047 | Mammals high |
| group_1221 | 0.071 | 0.047 | Mammals high |
| group_718 | 0.071 | 0.047 | Mammals high |


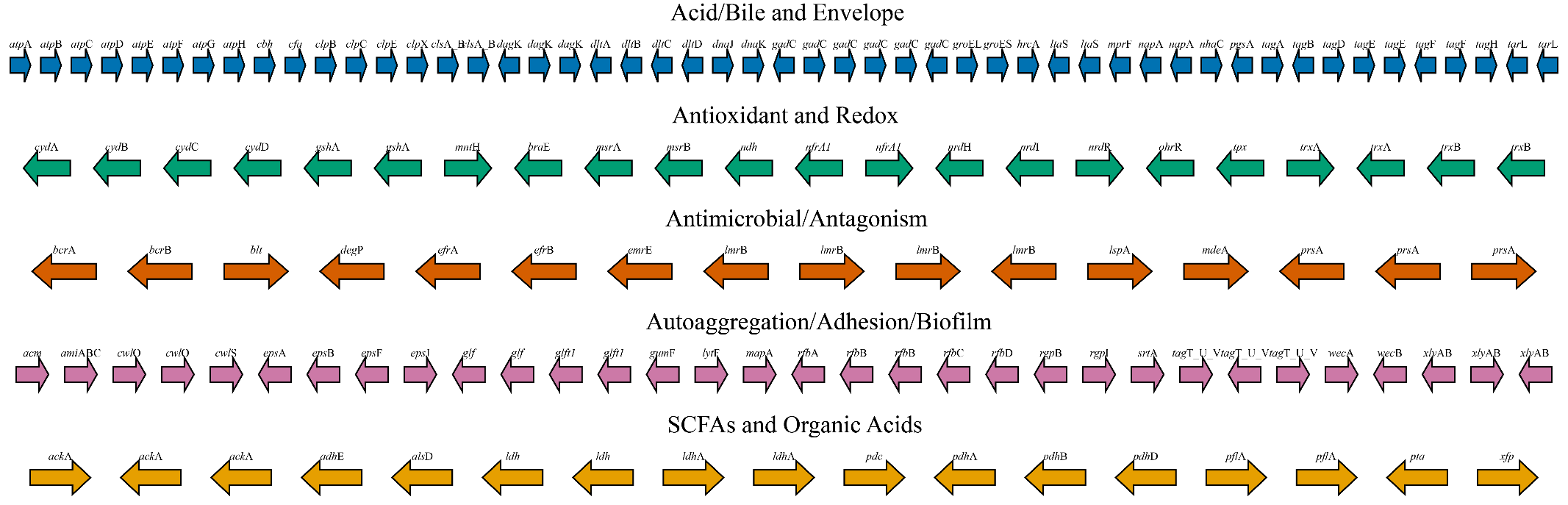


**Figure S2. Genomic organization of probiotic-associated genes in *L. agilis* 2-2.** Schematic representation of genes related to probiotic traits. Arrows indicate coding direction and are color-coded by function: blue, acid and bile tolerance and cell envelope biosynthesis; green, antioxidant and redox defense; orange, antimicrobial activity and competitive exclusion; pink, autoaggregation, adhesion, and biofilm formation; yellow, short-chain fatty acid and organic acid metabolism.
